# Perceived social support in solo women seeking treatment with donor gametes and in women in heterosexual couples seeking IVF-treatment with own gametes

**DOI:** 10.1038/s41598-023-29441-y

**Published:** 2023-02-15

**Authors:** Malin Lindell Pettersson, Gunilla Sydsjö, Claudia Lampic, Agneta Skoog Svanberg, Evangelia Elenis

**Affiliations:** 1 Department of Obstetrics and Gynecology, and Department of Biomedical and Clinical Sciences, Linköping University, SE-581 85 Linköping, Sweden; 2 Department of Psychology, Umeå University, 901 87 Umeå, Sweden; 3 Department of Women’s and Children’s Health, Uppsala University, Uppsala, Sweden

**Keywords:** Psychology, Health care, Risk factors

## Abstract

Solo motherhood is a family constellation that is becoming increasingly common in high income countries. The demographic characteristics of solo women entering treatment with donated sperm or embryo have been shown to be different from that of cohabiting women. The general importance of perceived social support is frequently amplified when health and quality of life are concerned, and positively affects mental health status, experienced stress, perceived self-efficacy during the transition to parenthood and during parenthood itself. The objective of the present study was to compare demographic characteristics, social network and perceived social support among solo women and cohabiting women awaiting fertility treatment. This objective was explored with a study-specific demographic and background questionnaire as well as through questions on access to practical support and the Multidimensional Scale of Perceived Social Support (MSPSS) assessing different sources of support. This study is a part of a longitudinal prospective multicenter study of solo women who awaited donation treatment in six Swedish public and private fertility clinics and a comparison group of women who were cohabiting/married to male partner and awaited in vitro fertilization (IVF) treatment with the couple’s own gametes. A total of 670 women were invited and 463 accepted participation (69% response rate); 207 solo women (study group) and 256 cohabiting women (comparison group). The results show significant differences in age, education, and employment between the groups. Solo women were on average 3.6 years older, had a higher level of education, a higher-income profession, and were more frequently working full time. Solo women perceived an equally high degree of social support from their families, significantly higher levels of support from friends and significantly lower support from a significant other compared to cohabiting women. Solo women expected their mother to be the most supportive person in future parenthood, while cohabiting women most often stated their cohabiting partner to fill that role. The study adds to the body of knowledge of solo women as a sociodemographic distinct group going at motherhood alone, stating a high degree of currently perceived and expected social support. The previously studied negative impact that lack of a co-parent might have, may be attenuated by the expected and perceived social support from family and friends.

## Introduction

Being a solo mother is a family constellation that is becoming increasingly common in high income countries. A single woman who opts to become a mother by use of sperm or embryo donation is referred to as solo mother, single mother by choice^1^, planned single mother or choice mother^2^. In the present study, they are henceforth referred to as “solo women”. Several studies have explored the motives and reasons behind going into parenthood alone among these women. The studies conclude that solo mothers often have a dream of a two-parent family but choose to become single mothers when they do not find a suitable partner to start a family with^3–6^ while feeling that their fertility is declining^5,7^. However, this is not always the case. One recent study found that some solo mothers considered being the only parent to be a perk since the woman would then be in control over parenting decisions without considering a co-parent’s opinion and without having to tend to the adult relationship^3^.

“Unplanned” single parenthood, i.e. due to separation, divorce, loss of spouse or in case of unplanned pregnancy when single, is a thoroughly investigated phenomenon both regarding the consequences for the child as well as for the single parent. In several Scandinavian countries and in the UK during 2018, approximately 20% of children below 17 years of age lived in a single-parent household while that number has been reported to be above 26%in the US^8^. Children growing up in single-parent households have been reported to have worse adjustment and prosocial behaviors when compared to children in two-parent families^9^ whereas single parents more often report financial stress, poor health and less social support^10,11^. However, single parenthood or the lack of a father/co-parent per se does not seem to pose a risk for maladjustment in the child. Rather, it seems like the increased risk of financial stress, poorer maternal mental health, lack of social support and prolonged conflicts between separated parents constitute major factors that affect the child living in a single-mother household^12,13^. In contrast, planned single parenthood by solo mothers has been shown to be related to equally good emotional and cognitive child adjustment as that seen among children of two-parent families^12,14–16^.

Social support, defined as the subjective belief or the objective help and assistance actually received by interacting with others, has been regarded crucial in order to cope with stressful events in life. In fact, *perceived* social support is highly correlated to psychological well-being, more so than for example tangible support^17^. The general importance of perceived social support is frequently amplified when health and quality of life are concerned^10,18,19^. Studies on the individual’s perceived social support during the transition into parenthood and during parenthood itself have underscored its crucial effect on the expectant parent’s mental health status, experienced stress, perceived self-efficacy and by extension the health and well-being of the child^20–26^. Higher levels of perceived social support have been shown to be associated with fewer reported depressive and anxiety symptoms by the parents, higher sense of competency^20^, fewer adverse effects due to low family income and parental depression^26^ as well as lower levels of parenting stress, ineffective parenting and child difficulties^24^.

In 2016, single women became eligible to request treatment with donated sperm in the Swedish public and private health care system. This possibility was previously exclusively offered to married/cohabiting women in heterosexual or homosexual relationships. In order for a solo woman to be eligible for donation treatment in Sweden, the woman has to undergo a thorough medical and psychosocial evaluation. The candidate for treatment needs to be unmarried and not cohabiting with a partner, of good physical and mental health, with a solid economic and housing situation, without criminal record, nor drug or alcohol abuse^27,28^. If the treatment is publicly funded, she must additionally be childless, below 40 years of age and meet specific BMI criteria (at or below 30 or 35 depending on clinic requirements) when entering treatment. Also, as for all treatments including donated gametes, the recipient needs to agree to the legal aspects of the donation which entails disclosing the use of donated gametes for conception to the future child^27–29^. Couples undergoing fertility treatments with their own gametes are not required to undergo a psychosocial assessment with regard to parenting adequacy. However, a recent study on access to ART in Sweden has demonstrated that the majority of ART candidates undergo some kind of assessment of their physical, psychological and social circumstances, regardless of whether the treatment involves the use of donated gametes or not^30^.

The existing studies on the ever-larger population of solo mothers are scarce and of relatively small samples and have been mainly conducted in countries where women self-fund their treatment. Furthermore, the primary focus in previous studies has been maternal health, psychological adjustment of the children and maternal-child relationship, while no study has explored the social circumstances surrounding solo mothers to be.

Support from a significant other aids the transition into motherhood^31^. How solo women who plan to pursue motherhood without a partner perceive current and expected support from their social network, and whether their perceptions differ from partnered women, has not been previously investigated. Thus, the aim of this study was to compare sociodemographic characteristics and perceived social support of solo women awaiting fertility treatment with donor sperm or embryos and cohabiting women awaiting in vitro* fertilization* (IVF) with the couple’s own gametes.

## Material and methods

### Study design, setting and participants

The current study is part of a longitudinal prospective multicenter project aiming to study solo women treated with gamete donation. The project’s objective is to enable proper care and support of these families within the maternal, child healthcare and child-care. Solo women, who sought and were accepted for donation treatment, and women who were cohabiting/married to a male partner and accepted for IVF treatment with the couple’s own gametes, henceforth referred to as cohabiting women, were invited to participate. The women in this sub-study were recruited during the time-period, April 2019 to March 2021 from the fertility clinics at the university hospitals in Uppsala, Örebro, Linköping, Malmö as well as Livio Fertility Centers in Stockholm and Falun. A total of 670 women accepted for and awaiting either self-funded or fully subsidized treatment were invited to participate, and 463 women accepted participation, yielding a study population of 207 solo women (study group) and 256 cohabiting women (comparison group), a response rate at 69% (71% and 68% respectively). Those who accepted to participate in the study, completed a survey questionnaire before treatment. The completed questionnaire was sent by air mail to the research group.

### Data collection

A study-specific demographic and background questionnaire was constructed and included questions on age, education, employment, and perceived financial hardship. Income was estimated using mean gross monthly income for the stated profession^32^. For assessment of social network, the participants were asked to state up to five of the most important persons they believed would be supportive in future parenthood. Participants were not explicitly instructed to rank the individuals that constituted their supportive network; however, an alphabetical order code was utilized for their responses (Person A-E). To facilitate interpretation and understanding of the results, only responses regarding Persons A-C are presented in the results section. The questionnaire entailed questions about the nature of the relationship to the person, the person’s age, employment status, the geographic distance to the person and to which extent she expected the person to be able to provide her with practical support.

The woman’s perceived social support was assessed using the Multidimensional Scale of Perceived Social Support (MSPSS) constructed by Zimet et al. in 1988^33^. The scale was developed as a short self-report instrument of subjectively assessed social support (i.e. perceived emotional and instrumental support) from family, friends and significant others respectively^33^ and consists of 12 items, rated on a 7-point Likert scale (from very strongly disagree to very strongly agree)^34^. MSPSS scores range from 1 to 7 where 1 means very strongly disagree and 7 means very strongly agree. The maximum mean score is 7. The MSPSS entails questions on perceived close and meaningful relations and assesses the perceived possibility to receive emotional and practical support, whether there is someone to share joys and sorrows with, if there is a special someone who cares about the respondent’s feelings and problems, and who helps her to make decisions^35^. The MSPSS has been translated to Swedish and evaluated in a Swedish context by Ekbäck et al. in 2013, and showed good validity and internal consistency where all MSPSS scales had a Cronbach’s alpha value equal to or greater than 0.9^36^.

### Statistical analyses

Categorical data are presented as number (n) and percent (%), while mean and standard deviation (SD) are used for continuous variables. Differences between groups on continuous variables were evaluated using Student’s *t* test. For group comparisons (study group and comparison group) regarding sociodemographic variables (e.g. level of education), social support (MPSS) and variables regarding most supportive persons (e.g. geographical proximity) Pearson’s Chi-square statistic or Fisher’s exact test was used, the latter when expected cell count was below 5. Analyses were performed using SPSS, version 28.0 (IBM Inc., Armonk, NY). Sample size calculation was performed for the entire longitudinal multi-outcome project, based on the clinical endpoint of preterm delivery (not explored in the present sub-study).

### Ethics approval and consent to participate

The study was approved by the Regional Ethical Review Board, Uppsala, Sweden (Dnr 2018/374 Date 07-11-2018) and was performed in accordance with the Declaration of Helsinki. All study participants have given oral and written informed consent.

## Results

### Socio-demographic characteristics

Solo women were more likely to be older compared to cohabiting women and were twice as often above 40 years of age (7.7% vs. 3.7%). More than 70% of the women in the comparison group were 34 years or younger, whereas almost 70% of the solo women were 35 years or older (Table 1). Further, solo women had a higher level of education compared to cohabiting women, with 80.8% and 63.3% respectively having attended university (Table 1).

**Table 1:** Socio-demographic background data on study participants, presented by group (solo women and cohabiting women).

	Solo women (n = 207)	Cohabiting women (n = 256)	p-value
	n (%)	n (%)	
Age			
–29 years	14 (6.8)	77 (30.1)	< 0.001
30–34 years	50 (24.2)	104 (40.6)	
35–39 years	127 (61.4)	66 (25.8)	
40–years	16 (7.7)	9 (3.7)	
Age, mean/SD^1^	35.65/3.57	32.04/4.20	< 0.001
University			
No	39 (18.8)	94 (36.7)	< 0.001
Yes	168 (81.2)	162 (63.3)	
Housing			
Single family home	21 (10.2)	123 (48.8)	< 0.001
Condominium	110 (53.4)	77 (30.6)	
Apartment	72 (35.0)	50 (19.8)	
Other	3 (1.5)	2 (0.8)	
Employment			
No	5 (2.4)	24 (9.6)	0.002
Yes	202 (97.6)	226 (90.4)	
Working full time			
No	17 (8.3)	43 (18.5)	0.002
Yes	189 (91.7)	189 (81.5)	
Monthly income*			
< 30,000 SEK	42 (20.3)	88 (37.6)	< 0.001^2^
30,000–44,700 SEK	139 (67.1)	129 (55.1)	
> 44,700 SEK	26 (12.6)	17 (7.3)	
Self-reported financial hardship			
No	203 (99.0)	242 (96.8)	0.197^3^
Yes	2 (1.0)	8 (3.2)	

^1^Student’s *t* test.

^2^Income estimated according to occupation.

^3^Fisher’s exact test.

Regarding rate of (un)employment almost 10% of women in the comparison group were either students, unemployed or on sick/parental leave whereas 97.6% of solo women were employed, with the majority of them working full time. Moreover, 67% of solo women and 55% of cohabiting stated middle income professions (Table 1). Solo women reported a higher monthly income and were almost twice as likely to have an earned income above 44,700 SEK/ ~ 5400 USD/ ~ 4400 Euro while cohabiting women were twice as likely to have an income below 30,000 SEK/ ~ 3600 USD/ ~ 3000 Euro per month. (Table 1). Nevertheless, when asked about their perception of their economic situation and whether they were in financial hardship, women did not differ regarding their responses.

Concerning who they regarded to be part of their close family, significantly more solo women compared to cohabiting women included their mother/stepmother (95.7% vs. 85.8% respectively), followed by their father/stepfather (86.5% vs. 76.3% respectively) and lastly by siblings/step siblings (64.5% vs. 55.3% respectively). Finally, 14.7% of cohabiting and 3.4% of solo women reported they already had biological children while 5% in the former group and 1% of the latter reported they had stepchildren (Data not shown).

### The multi-dimensional scale on perceived social support

Concerning MSPSS both groups reported a similarly high mean total score (6.40 and 6.44 out of 7) reflecting an overall high degree of current perceived social support (Table 3). Group comparisons of sub-scale scores showed significant differences, with solo women reporting higher levels of support from friends and lower support from a significant other than did cohabiting women. The level of perceived social support from family was equal between the study and comparison group (Table 2).

**Table 2:** Mean scale scores on The Multidimensional Scale of Perceived Social Support (MSPSS) sub-scales and total score^1,2^.

	Solo women (n = 256)	Cohabiting women (n = 207)	p-value	t-value [df]	Cohen’s d
	Mean/SD	Mean/SD			
Total score	6.44/0.61	6.40/0.65	0.538	− 0.617 [443]	0.62985
Significant other	6.60/0.63	6.84/0.42	< 0.001	4.639 [336]	0.52493
Family	6.31/0.91	6.30/0.92	0.856	− 0.181 [439]	0.91467
Friends	6.42/0.77	6.09/1.09	< 0.001	− 3.599 [454]	0.95781

^1^All scales ranges between 1 and 7.

^2^Student’s t-test.

### Important persons expected to provide support in future motherhood

The vast majority of solo women (89.4%) reported that the first (Person A) among the most important individuals to support her in her role as a mother would be a female, either a parent (71%) or a sibling (15.5%), while for cohabiting women person A was primarily their male partner (82.7%). Five solo women stated that the first person was a (sexual) partner (2.4%).

Women from the comparison group (94.5%) declared that Person A was cohabiting or living closely to them, while for 27% of the solo women that person lived more than an hour’s drive away or abroad. Solo women’s first person was equally likely to be employed full time or to be retired, while for 86.2% of the cohabiting women that person worked full-time. Regarding the expectations on practical support (such as regarding childcare) from their first reported person 86.6% of cohabiting women reported a high or very high degree of expected support while only two thirds of the solo women had equivalent expectations (Table 3).

**Table 3:** Socio-demographic data on the three most important persons the participants expect to provide support in future parenthood^1,2^.

	Person A					Person B					Person C				
	Solo women n = 207	Cohabiting women n = 256	p-value	Chi-square	Phi	Solo women	Cohabiting women	p-value	Chi-square	Phi	Solo women	Cohabiting women	p-value	Chi-square	Phi
	n (%)	n (%)				n (%)	n (%)				n (%)	n (%)			
Gender															
Man	22 (10.6)	213 (84.2)	< 0.001	246.548	0.732	106 (51.7)	41 (16.6)	< 0.001	62.920	− 0.373	71 (34.5)	97 (39.9)	0.234	1.415	0.056
Woman	185 (89.4)	40 (15.8)				99 (48.3)	206 (83.4)				135 (65.5)	146 (60.1)			
Relation															
Partner	5 (2.4)	210 (82.7)	< 0.001	359.961	0.808	0 (0.0)	10 (4.0)	0.001	20.928	0.209	2 (1.0)	8 (3.2)	< 0.001	70.565	0.390
Parent	147 (71.0)	35 (13.8)				118 (57.0)	163 (65.2)				36 (17.4)	107 (43.3)			
Sibling	32 (15.5)	4 (1.6)				48 (23.2)	38 (15.2)				90 (43.5)	39 (15.8)			
Friends	19 (9.2)	1 (0.4)				30 (14.5)	20 (8.0)				58 (28.0)	46 (18.6)			
Other family	1(0.5)	2 (0.8)				8 (3.9)	17 (6.8)				18 (8.7)	45 (18.2)			
Age															
< 30	4 (1.9)	43 (17.0)	< 0.001	180.229	0.626	5 (2.4)	14 (5.6)	< 0.001	26.750	0.242	21 (10.2)	20 (8.2)	< 0.001	67.275	0.386
30–39	33 (15.9)	136 (53.8)				59 (28.5)	47 (18.8)				108 (52.7)	63 (25.8)			
40–49	20 (9.7)	38 (15.0)				18 (8.7)	14 (5.6)				33 (16.1)	19 (7.8)			
50–59	20 (14.0)	20 (7.9)				26 (12.6)	73 (19.2)				11 (5.4)	51 (20.9)			
60–69	99 (47.8)	12 (4.7)				71 (34.3)	82 (32.8)				25 (12.2)	75 (30.7)			
70–^3^	22 (10.6)	4 (1.6)				28 (13.5)	20 (8.0)				7 (3.4)	16 (6.6)			
Geographic distance															
Cohabitant	6 (2.9)	208 (81.9)	< 0.001	289.037	0.794	0 (0.0)	12 (4.8)	0.002	16.010	0.178	0 (0.0)	8 (3.3)	0.001	18.029	0.197
Walking distance	83 (40.7)	9 (3.5)				82 (40.0)	73 (29.1)				83 (40.7)	73 (29.7)			
Shorter route	60 (29.4)	23 (9.1)				69 (33.7)	92 (36.7)				61 (29.9)	107 (43.5)			
Longer route/abroad	55 (27.0)	14 (5.5)				54 (26.3)	74 (29.5)				60 (29.4)	58 (23.6)			
Occupation															
Full-time	84 (41.0)	219 (86.2)	< 0.001	120.916	0.513	105 (51.2)	148 (59.2)	0.309	3.596	0.089	128 (62.7)	147 (59.5)	0.005	12.794	0.168
Part-time	23 (11.2)	15 (5.9)				17 (8.3)	19 (7.6)				19 (9.3)	34 (13.8)			
Unemployed/other^4^	14 (6.8)	12 (4.7)				15 (7.3)	19 (7.6)				33 (16.2)	19 (7.7)			
Retiree	84 (41.0)	8 (3.1)				68 (33.2)	64 (25.6)				24 (11.8)	47 (19.0)			
Expected degree of support															
Very/quite low	25 (12.2)	20 (7.9)	< 0.001	28.431	0.249	49 (23.9)	68 (27.2)	0.463	1.538	0.058	63 (30.7)	86 (34.8)	0.015	8.452	0.137
Quite high	42 (20.5)	14 (5.5)				60 (29.3)	61 (24.4)				78 (38.0)	63 (25.5)			
High/very high	138 (67.3)	220 (86.6)				96 (46.8)	121 (48.4)				64 (31.2)	98 (39.7)			

^1^The number does not always add up to the group totals (207 and 256 respectively) due to partially missing values.

^2^Pearson’s Chi-square or Fisher’s exact test (when cell count was below 5).

^3^Oldest person A = 75, oldest person B = 79, oldest person C = 84.

^4^On sick leave, on parental leave or student.

The second person (Person B) reported by both solo women and cohabiting women was most often either a parent, a sibling, or a friend (57%, 23.2%, 14.5% versus 65.2%, 15.2% and 8% respectively). For most cohabiting women the second person was of female gender (83.4%) while for solo women that person was equally often of male or female gender (Table 3).

## Discussion

Comparison of solo women who were accepted for gamete donation treatment and cohabiting women awaiting standard IVF treatment revealed that solo women were older with a higher socioeconomic status. They perceived an overall equally high degree of social support as cohabiting women while scoring higher regarding support from friends. Solo women expected a great deal of support in their motherhood from their nearby living, retired or full-time working parents (or stepparents) and secondarily, from a female friend.

The present results on the socioeconomic status of solo women are, in line with previous research^4,5,7,37^. Indeed, several studies have reported that solo women had made careful plans regarding education, work, economy and social network before pursuing solo motherhood^3,7^. This may account for their healthy finances but might actually have delayed them from seeking help and undergoing fertility treatment earlier. Furthermore, as the change of the Swedish legislation allowing solo women to pursue fertility treatment with donated sperm and embryos took place quite recently (i.e. in 2016 and 2019 respectively), it is possible that some of the solo women would have sought treatment at a younger age had it been available within the Swedish borders. The present finding regarding their more advanced educational level and higher income is not surprising since solo women in Sweden are evaluated regarding their socio-economic status and only considered eligible for treatment with donated sperm or embryos if they fulfill certain criteria^30^. While there is no corresponding mandatory psychosocial evaluation for heterosexual couples seeking IVF-treatment with their own gametes in Sweden, a recent study showed that all forms of assisted reproductive treatments could be denied or delayed due to factors believed to entail a risk or harm to the health of the future child, such as financial or occupational constraints^30^. Thus, it is possible that cohabiting women participating in our study had also undergone an evaluation of the couple’s socioeconomic situation. The decision to grant treatment would therefore be based not solely on their personal status but on their joint status which in turn may partly explain the reported differences in occupation and income level between the women in the study and the comparison group (e.g. a fourfold proportion of women in the comparison group reported being unemployed, students or on sick leave compared to solo women).

Regarding solo women’s perception of social support, their total score was high on the MSPSS scale and similar to that of cohabiting women. For as many as 71% of the solo women in the present study, the first person expected to be supportive in their life as mothers was their parent (most often their mothers). This result is in line with the findings of Werner et al.^7^ exploring solo mother’s effort to ensure social support and back-up from family and friends before the fertility treatment^7^. As the average lifespan increases, contemporary grandparents are healthier, live longer and are more available, playing an important role as caretakers^38^. According to a UK based study, grandparents might even take on the role of a replacement partner by attending to the practical everyday needs of their adult daughter who is a solo mother, or a replacement parent by taking on a fostering role to the grandchild^39^. Further, transition to grandparenthood has been associated with better well-being of the grandparents, especially during the early period of grandparenthood^40^, which also coincides with the first vulnerable year of motherhood. As satisfaction with the role of grandparent positively correlates to the degree of closeness between the child and the grandparent^41^, grandparent involvement might actually positively affect the relationship between the child, mother and grandparent. Grandparents’ greater involvement could to some extent attenuate potential differences between the families of solo women and cohabiting women and make up for the lack of a co-parent.

Solo women reported a significantly higher level of social support from friends compared to cohabiting women. The importance of social support from friends has been apparent in previous studies on both single and cohabiting mothers^42–44^. Luthar and Ciciolla^44^ found that support from friends was positively associated with life satisfaction and fulfilment and negatively linked to stress, emptiness, depression, anxiety and loneliness in a sample of 2000 single and married mothers^44^. Thus, social support from friends may constitute an important buffer for emotional distress in solo women. However, such support also entails an implicit expectation of reciprocity, in contrast to received support from a family member^11,45^.

Solo women differed from cohabiting women concerning the degree of practical support expected from their most important close persons. Prior studies on solo mothers repeatedly report that solo women are reluctant to ask for help from their support network or healthcare providers even when in need^7,46,47^. They do not wish to burden other people or society since it was their deliberate choice to become single parents. This attitude could compromise both the extent to which social support will be actively sought by the solo mother and the extent to which social support in fact will be provided by the grandparents and friends. It is also important to underscore that advanced maternal age (defined as ≥ 35 years of age) has been associated with several adverse obstetric outcomes^48–52^, which in turn could increase these women’s need of support from both friends and family, and the welfare system.

An unexpected finding was that a small number of solo women responded that they expected to receive practical support in their parenthood from a partner (boyfriend/girlfriend/ or non-cohabiting partner). Unfortunately, further information on why these women decided to become single candidates for parenthood despite having a partner at the time they underwent the medical and psychosocial evaluation was not available. Fear of rejection due to partner’s circumstances or reluctance to wait for the necessary time duration in order to prove relation stability could be reasons for applying as single instead of as cohabiting women. The finding could also reflect that they entered a relationship after being accepted for treatment. That could potentially make the interpretation of study results more challenging but should not affect our findings as a whole.

The large sample size and the relatively high response rate (69%) are considered satisfactory for a survey and mark a high interest from both solo and cohabiting women to participate in the study. Still, a non-response rate of 31% could potentially compromise the generalizability of the findings and reflect a selection bias^53^. Lastly, women reporting on the expected future support from specific persons in their social network were not explicitly instructed to rank these persons according to the expected degree of support, which constitutes a limitation of the study. However, the alphabetical sorting code used for reporting (Person A-E) implies an inherent form of ranking. The responses received indicate that this is also how the question generally was perceived by both the study and comparison group as participants most often reported the individuals expected to provide a high degree of support (very high/high) in the positions A-C, with the vast majority of cohabiting women reporting first their male partner (e.g. position A). We can therefore safely assume that the response order was not given by chance alone.

Solo women awaiting gamete donation treatment within a tax-funded health care system generally have a high socioeconomic status with regard to occupational attainment and financial security. They perceive themselves well equipped with social support and report significantly higher levels of support from friends compared to women pursuing IVF-treatment as part of a heterosexual couple. While most cohabiting women rely on their partner as the primary source of support in future parenthood, solo women expect to receive substantial help and support primarily from their parents and female friends. However, requesting support from friends and family has shown to be associated with feelings of shame among solo women, and assumptions of reciprocity could further complicate the actual use of available support. Therefore, future studies are required to evaluate to what extent solo women request and receive the level of social support that they expect from their social network once the baby is born. These studies are of great importance to evaluate and to ensure maternal and child well-being in future solo-mother families.

## Data Availability

The dataset used in the current study is available from the corresponding author on reasonable request.
